# ﻿DNA barcoding of passerine birds in Iran

**DOI:** 10.3897/zookeys.1236.143336

**Published:** 2025-04-24

**Authors:** Sahar Javaheri Tehrani, Elham Rezazadeh, Niloofar Alaei Kakhki, Leila Nourani, Vali Ebadi, Sahar Karimi, Mojtaba Karami, Fatemeh Ashouri, Asaad Sarshar, Toni I. Gossmann, Mansour Aliabadian

**Affiliations:** 1 Department of Biology, Faculty of Science, Ferdowsi University of Mashhad, Mashhad, Iran TU Dortmund University Dortmund Germany; 2 Computational Systems Biology, Faculty of Biochemical and Chemical Engineering, TU Dortmund University, Dortmund, Germany Ferdowsi University of Mashhad Mashhad Iran; 3 Department of Animal Biology, Faculty of Biological Sciences, Kharazmi University, Tehran, Iran Kharazmi University Tehran Iran; 4 Department of Zoology, State Museum of Natural History Stuttgart, Stuttgart, Germany State Museum of Natural History Stuttgart Stuttgart Germany; 5 Department of Environmental Sciences, Faculty of Natural Resources and Environment, Malayer University, Malayer, Iran Ferdowsi University of Mashhad Mashhad Islamic Republic of Iran; 6 Research Department of Zoological Innovations, Institute of Applied Zoology, Faculty of Science, Ferdowsi University of Mashhad, Mashhad, Iran Malayer University Mashhad Islamic Republic of Iran

**Keywords:** *COI* gene, Genetic diversity, Selection, Species delimitation

## Abstract

Exploring genetic diversity is essential for precise species delimitation, especially within taxonomically complex groups like passerine birds. Traditional morphological methods often fail to resolve species boundaries; however, DNA barcoding, particularly through the mitochondrial cytochrome c oxidase subunit I (*COI*) gene, provides a powerful complementary method for accurate species identification. This study establishes a comprehensive DNA barcode library for Iranian passerine birds, analyzing 546 *COI* sequences from 94 species across 23 families and 53 genera. There is a pronounced barcode gap, with average intraspecific divergence at 0.41% and interspecific divergence at 18.6%. Notable intraspecific variation emerged in the Persian nuthatch (*Sittatephronota*) and the Lesser whitethroat (*Currucacurruca*), while the European goldfinch (*Cardueliscarduelis*) and the grey-crowned goldfinch (*Cardueliscaniceps*) showed limited genetic differentiation despite marked morphological distinctions. Phylogenetic analysis revealed significant east-west genetic splits in *C.curruca* and *S.tephronota*, reflecting Iran’s geographic and zoogeographic boundaries. These findings demonstrate the effectiveness of DNA barcoding in elucidating biogeographic patterns, emphasizing Iran’s key role as an ornithological crossroads for avian biodiversity. Moreover, our results suggest that much of the genetic variation in the *COI* gene arises from synonymous mutations, highlighting the role of purifying selection in shaping mtDNA diversity across species.

## ﻿Introduction

Genetic diversity is a fundamental aspect of biodiversity, representing the variety of genetic information within and among species (Nonić and Šijačić-Nikolić 2021). This genetic diversity plays a critical role in evolutionary processes such as natural selection and adaptation, driving speciation and shaping the phylogenetic relationships among organisms (Huang et al. 2016; Nonić and Šijačić-Nikolić 2021). Accurate assessment of genetic diversity is also fundamental for species delimitation, particularly in cryptic or morphologically similar species, where traditional taxonomic methods may fall short (Hebert et al. 2003; Lohman et al. 2009; Bilgin et al. 2016). Different genetic markers offer varying effectiveness in studying genetic diversity and it is recommended to use fast changing molecular markers (i.e., coding vs noncoding DNA) for closely related species (Abdel-Mawgood 2012). DNA barcoding is a transformative technique in biodiversity research, allowing for the precise identification and differentiation of species through genetic markers. This method utilizes a standardized, short segment of the mitochondrial gene cytochrome c oxidase I (*COI*), typically a 648-base pair region, to serve as a species tag for species identification and delimitation (Arida et al. 2021; Zhang and Bu 2022). This species delimitation relies on DNA barcoding gap, which refers to the difference between mean intraspecific and interspecific genetic distances (Antil et al. 2023) and beyond taxonomy (Chac and Thinh 2023), this approach has also been employed in studies of biogeography, ecology and biological conservation (Gostel and Kress 2022; Wu et al. 2023). Moreover, genetic diversity levels in mitochondrial DNA are influenced by various factors, primarily mutation rate, selection, and effective population size (Clark et al. 2023). Understanding the selective forces acting on *COI* sequences provides valuable insights into species evolutionary histories and adaptive responses, with important implications for biodiversity conservation strategies and for tracking ecological changes over time (Matzen da Silva et al. 2011).

Birds represent one of the most extensively studied animal groups in DNA barcoding projects (Hebert et al. 2004), achieving species-level identification accuracy ranging from 93 to 99% (Colihueque et al. 2021). This high level of accuracy demonstrates the effectiveness of DNA barcoding in discriminating among sympatric avian species (Colihueque et al. 2021). While the majority of DNA barcoding studies on avian diversity has been concentrated in Europe and North America (Hebert et al. 2004; Johnsen et al. 2010; Bilgin et al. 2016), significant efforts have also been undertaken in other regions, such as the Neotropics (Kerr et al. 2009a, b; Tavares et al. 2011), South Korea (Yoo et al. 2006), eastern Palearctic (Kerr et al. 2009b), Indomalaya (Lohman et al. 2009; Lohman et al. 2010), and Australasia (Patel et al. 2010). Consequently, DNA barcodes are currently available for approximately 41% of bird species worldwide, encompassing approximately 4,300 species from 37 of the 39 recognized avian orders (Colihueque et al. 2021). Despite the expansion of DNA barcode reference databases in species diversity and geographic coverage (Gostel and Kress 2022; Cheng et al. 2023), many regions remain underrepresented, resulting in a notable geographic bias in barcoded species representation (Colihueque et al. 2021). This gap highlights an urgent need for more comprehensive sampling in these poorly documented areas (Gostel and Kress 2022). Furthermore, obtaining the required permits for specimen collection and transporting samples across national borders is often particularly complex, especially for birds (Lijtmaer et al. 2012), which adds to the challenges addressing biodiversity gaps.

Iran is recognized as a globally significant biodiversity hotspot, characterized by its remarkable species richness and high levels of endemism (Aliabadian et al. 2005, 2007; Noori et al. 2024; Rezazadeh et al. 2024). This diversity can be largely attributed to the country’s geographic complexity, steep climatic gradients, and pronounced landscape heterogeneity (Noori et al. 2024). Additionally, Iran’s unique geographic position serves as a zoogeographical transition zone where several major biogeographical realms—the Palearctic (both eastern and western), Oriental, and Afrotropical—intersect (Noori et al. 2024). This geographic positioning not only establishes Iran as an ornithological crossroads but also contributes to the notable presence of sister bird species within the country (Aliabadian et al. 2005). However, despite constituting a significantly more important hotspot for diversity, the population structure and genetic diversity of the passerine taxa—representing the most species-rich clade of birds—remain inadequately explored within the country. To address this gap, we have conducted an extensive sampling effort to generate a comprehensive DNA barcoding library of Iranian Passerine birds.

Our main objectives are (i) to evaluate genetic variation in *COI* among passerine birds in Iran—a region characterized by numerous contact zones between passerine species (Aliabadian et al. 2007)—to provide new insights into the efficacy of *COI*-based DNA barcoding; (ii) to identify potential cryptic species; and (iii) to investigate the impact of natural selection on mitochondrial *COI* sequences.

## ﻿Materials and methods

### ﻿Taxon sampling

The study area covers the northeastern and western regions of Iran (Suppl. material 1: fig. S1). We examined 546 individuals representing 94 species from all these regions, with 75 species of these taxa (80% of species) represented by more than two individuals. All birds were captured during breeding season using mist nets, identified, and then released following the collection of feather and blood samples. Blood samples were collected from the brachial vein of each bird following standard protocols and preserved in Queen’s buffer (Seutin et al. 1991). No birds were harmed during the capture, handling, and blood collection process. For the taxonomy of species, we used the IOC World Bird List v. 14.2 (Gill et al. 2024). The complete list of sampled specimens including information about geographical location, voucher and access numbers are provided in Suppl. material 1: table S1.

### ﻿Laboratory procedures

DNA was extracted from blood and feather samples using a standard salt extraction method (Bruford et al. 1992), following overnight incubation at 40 °C in an extraction buffer containing 2% sodium dodecyl sulfate (SDS) and 0.5 mg/ml proteinase K. Additionally, 30 µl DTT was added during the initial incubation step for feather extraction. The COI gene was selected as the molecular marker of choice, as it is widely recognized, with more than 3,000 papers published on the application of COI barcodes for the identification and discovery of animal species (Pentinsaari et al. 2016). Primer pairs and a locus-specific annealing temperature that have been used to amplify this gene region are shown in Table 1. Total PCR reaction volumes were 25 μl, containing 12.5 μl Taq DNA Polymerase Master Mix RED (Ampliqon), 1 μl of each primer with a concentration of 10 μM, 3 μl DNA, and 7.5 μl ddH2O. PCR products were examined on 2% agarose gels to confirm the successful amplification of the target fragments. The purified PCR products for all specimens were sequenced by Macrogen Inc (Seoul, South Korea).

**Table 1.** Primer pairs that have been successfully used to obtain bird barcodes. This table includes forward and reverse primer names, primer sequences, annealing temperature, and citation.

Primer Name	Primer Sequences (5‘-3‘)	Annealing Temperature	Citation
**BirdF1**	TTCTCCAACCACAAAGACATTGGCAC	50 °C	(Johnsen et al. 2010)
**BirdR1**	ACGTGGGAGATAATTCCAAATCCTG	50 °C	(Johnsen et al. 2010)
**BirdR2**	ACTACATGTGAGATGATTCCGAATCCAG	50 °C	(Johnsen et al. 2010)
**PasserF1**	CCAACCACAAAGACATCGGAACC	58 °C	(Lohman et al. 2009b)
**PasserR1**	GTAAACTTCTGGGTGACCAAAGAATC	58 °C	(Lohman et al. 2009b)
**AWCF1**	CGCYTWAACAYTCYGCCATCTTACC	57.5 °C	(Patel et al. 2010)
**AWCR6**	ATTCCTATGTAGCCGAATGGTTCTTT	57.5 °C	(Patel et al. 2010)

### ﻿Data analysis

Sequences were aligned and edited in BIOEDIT v. 7.0.1 (Hall 1999). Intraspecific and interspecific distances were calculated using Kimura 2-parameter (K2P) pairwise genetic distances in MEGA v6.0 (Tamura et al. 2013). Average intraspecific distances were determined for species with at least two sequences using MEGA. The K2P model was employed for all sequence comparisons, as it is considered the most effective metric for evaluating closely related taxa (Nei and Kumar 2000; Aliabadian et al. 2013; Carew and Hoffmann 2015). The best-fit model was estimated using JMODELTEST v. 2.1. (Darriba and Posada 2014) based on the Bayesian Information Criterion (BIC). The best model was then used to construct a phylogenetic tree with MRBAYES v. 3.2.0 (Ronquist and Huelsenbeck 2003) to provide a general graphic representation of the pattern of divergence between all species. Bayesian Inference (BI) analysis was carried out using the Markov chain Monte Carlo (MCMC) convergence implemented in MRBAYES v. 3.2.0. Two independent runs with four chains were run simultaneously for five million generations, with trees and parameters subsampled every 1000 generations. The first 50,000 trees (as a conservative ‘burn-in’) were discarded. Posterior probabilities (PP) were calculated from the remaining trees using a majority-rule consensus analysis.

The phylogenetic tree was rooted with one representative of Galliformes (*Gallusgallus*). Two criteria used for identifying and confirming species based on their DNA barcode if: a) it was monophyletic (i.e., the species formed a single cluster) and b) it did not share a barcode with any other species. Consequently, high intraspecific genetic distances in the *COI* gene are frequently utilized to predict cryptic or potentially new species. In our dataset, this pattern is observed in two species showing elevated genetic distances: the Persian Nuthatch *Sittatephronota* Sharpe, 1872 and the Lesser Whitethroat *Currucacurruca* Linnaeus, 1758 (Suppl. material 1: table S2). Our dataset includes the Goldfinch *Cardueliscarduelis* Linnaeus, 1758 and Grey-crowned Goldfinch *C.caniceps* Vigors, 1831, which hybridize in their contact zone in Iran (Haffer 1977) but have recently been recognized as two full species (Gill et al. 2024). Some members of the *C.curruca* complex may also represent full species (Abdilzadeh et al. 2023), warranting further investigation. For these three taxa, a Neighbor-Joining (NJ) tree was constructed using K2P distances in MEGA. This analysis incorporated the sequences obtained in this study and those deposited in BOLD (https://www.barcodinglife.org) from Iran. Furthermore, a haplotype network was implemented in POPART v. 1.7 (Leigh et al. 2015) to visualize the relationships among haplotypes. Pairwise K2P distances between populations were estimated with the program MEGA, and pairwise F_ST_ values were calculated with DNASP v. 5.1 (Librado and Rozas 2009)

### ﻿Coding DNA genetic diversity analysis

Based on the *COI* sequence fragments and the subsequent global alignment we obtained genetic diversity at 0-fold and 4-fold sites for all species. For this, we used the vertebrate mitochondrial genetic code with MEGA. We then used the Tajimas_d package from the bfx suite (https://py-bfx.readthedocs.io/en/latest/) to calculate nucleotide diversity for each species for 0-fold and 4-fold sites, respectively. We excluded species with zero diversity for either 4-fold or 0-fold sites. One can quantify effective population by dividing genetic diversity with the mutation rate per generation (π = 2Ne * µ, where Ne equals the effective population size, µ, the mutations per generation and π is the observed pairwise differences in a population genetic sample). Because we have limited knowledge of mitochondrial gene specific mutation rates for all passerine birds and only rough estimates for generation time, we cannot directly estimate effective population sizes. However, here we use genetic diversity at silent sites as a proxy for effective population size, which is not unreasonable because we restrict our analysis to passerine birds, a taxonomic group with supposedly little variation in mutation rate and generation time (Nguyen and Ho 2016).

## ﻿Results

### ﻿COI sequence variation

**Figure 1.**
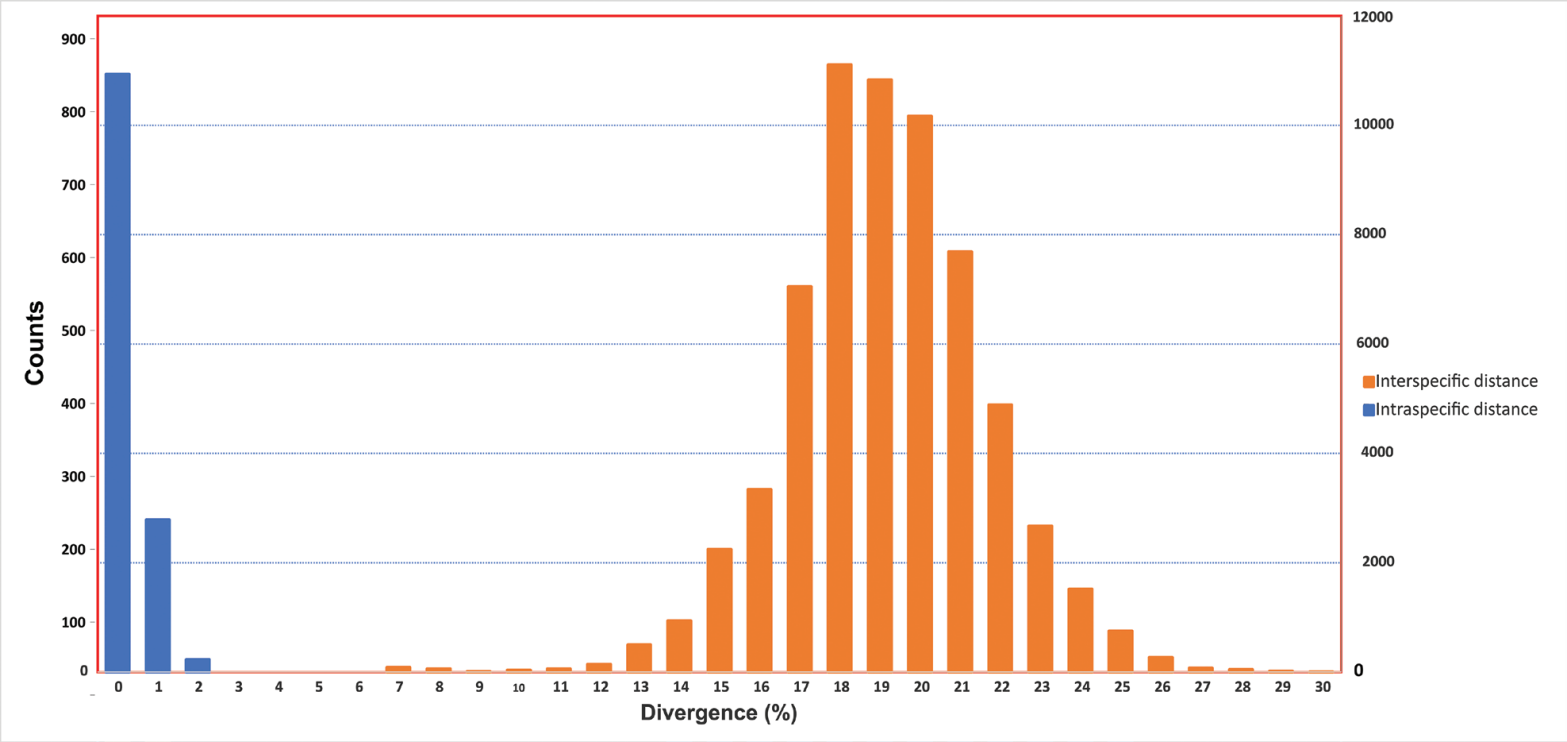
Comparisons of K2P pairwise distances based on the *COI* gene of 96 passerine bird species. Intraspecific distances are indicated with blue bars and interspecific distances with orange bars. Left Y-axis: numbers of intraspecific comparisons; right Y-axis: numbers of interspecific comparisons.

A total of 546 sequences from 94 passerine bird species were generated and uploaded to the NCBI database (publicly available, Suppl. material 1: table S1), representing 53 different genera and 23 families of Passeriformes. We reconstructed a phylogenetic tree with a Bayesian approach using all the specimens (546 sequences, Suppl. material 1: fig. S2) to provide an initial overview of the species in our dataset. Most nodes in the resulting tree were well resolved and strongly supported with posterior probability (PP) more than 95%, as indicated by bold lines in the tree. Based on our dataset, of the 23 families included in the tree, 20 had high nodal support (≥ 0.95) and three had low nodal support (including Muscicapidae, Emberizidae and Fringillidae). Similarly, 51 of the 53 genera (excluding *Emberiza* and *Luscinia*) appeared monophyletic. Furthermore, all species formed well-supported (PP > 95%) monophyletic groups except *Laniuscollurio* which was not monophyletic (i.e., paraphyly occurs) (Suppl. material 1: fig. S2). The mean number of sequences per species was six (1–32). The mean intraspecific K2P distance was 0.41% (range: 0–3.61%), while the mean interspecific distance was 18.6% (range 0.17–29.64%) (Fig. 1). Notably, two species exhibited relatively high intraspecific divergence: *Sittatephronota* (2.30%) and *Currucacurruca* (3.60%).

**Figure 2.**
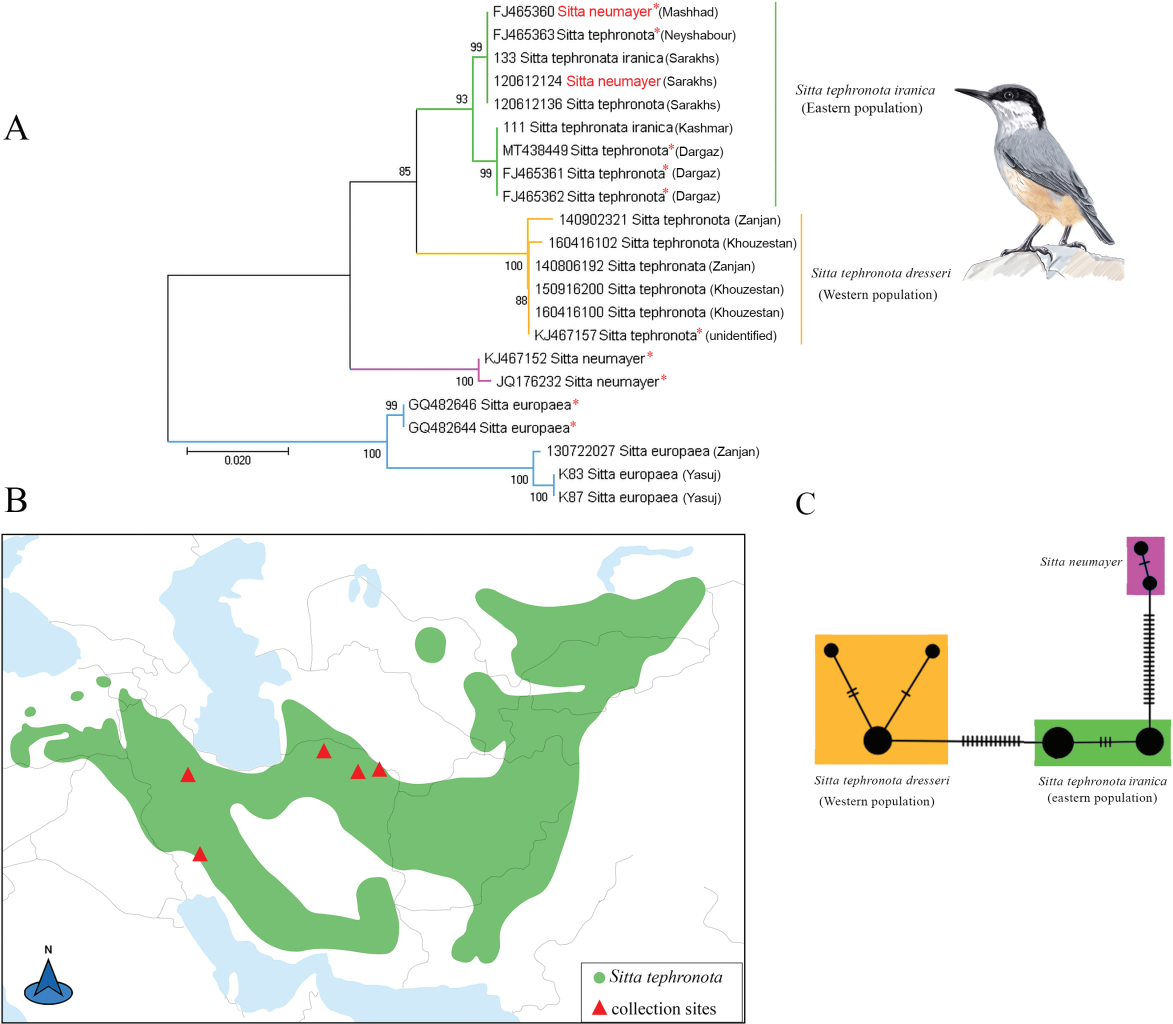
Phylogenetic and haplotype network analysis of *COI* data for *S.tephronota* and the origin of study material **A** neighbor-joining tree, values on the branches shows bootstrap values and, an asterisk indicates Iranian *COI* sequences from GenBank **B** distribution range and collection sites for the samples included in the study. Distribution map of *S.tephronota*, with green indicating areas where the species is native resident according to bird species distribution maps of the world (https://datazone.birdlife.org); sampling sites are indicated by red triangles **C** haplotype network, where colors indicate the origin of the haplotypes (orange: western population; green: eastern population; blue: *S.neumayer*) and the number of bars at each branch indicates the number of mutations.

**Figure 3.**
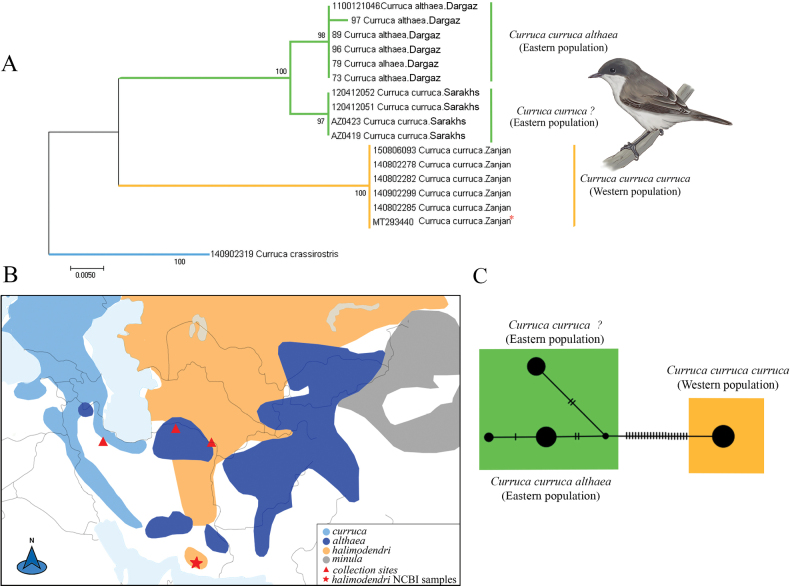
Phylogenetic and haplotype network analysis of *COI* data for *C.curruca* and the origin of study material **A** neighbor-joining tree, values on the branches shows bootstrap values and, an asterisk indicates Iranian *COI* sequences from GenBank **B** distribution range and collection sites for the samples included in the study. Approximate presumed breeding ranges of *C.curruca* taxa, modified from the map by Abdilzadeh et al. 2023; sampling sites are indicated by red triangles and GenBank sequences of *C.c.halimodendri* by a red star **C** haplotype network, where colors indicate the origin of the haplotypes (Orange: western population; Green: eastern populations) and the number of bars at each branch indicates the number of mutations.

**Figure 4.**
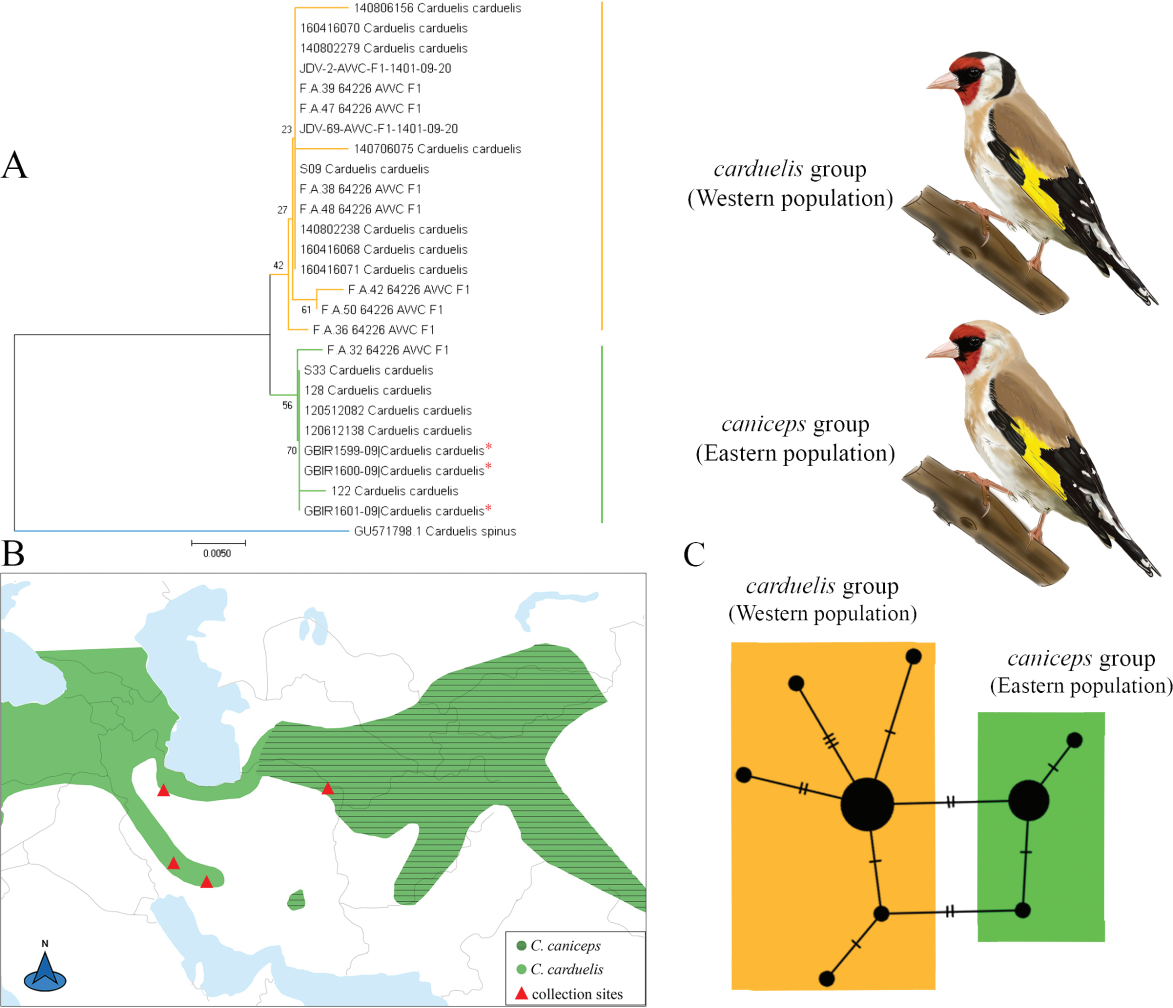
Phylogenetic and haplotype network analysis of *COI* data for *C.carduelis*, *C.caniceps* and the origin of study material **A** neighbor-joining tree, values on the branches shows bootstrap values and, an asterisk indicates Iranian *COI* sequences from GenBank **B** distribution range and collection sites for the samples included in the study. Distribution map of *C.carduelis* and *C.caniceps*, with green indicating areas where the species is native resident according to bird species distribution maps of the world (https://datazone.birdlife.org); sampling sites are indicated by red triangles **C** haplotype network, where colors indicate the origin of the haplotypes (orange: western species; green: eastern species) and the number of bars at each branch indicates the number of mutations.

Conversely, our analyses revealed low interspecific genetic distance between *Cardueliscarduelis* and *Cardueliscaniceps* (0.71%), which contrasts with the significant morphological differentiation observed (Suppl. material 1: table S2). In order to illustrate the basic pattern in these taxa, their results were presented in detail in Figs 2–4, respectively.

### ﻿Deep and shallow intraspecific divergences

*Sittatephronota* includes three subspecies in Iran, i.e., *S.t.dresseri* (Zagros Mts. in SE Turkey to N Iraq and W Iran), *S.t.obscura* (NE Turkey to the Caucasus and Iran) and *S.t.iranica* (NE Iran and S Turkmenistan). For this species, we analyzed six samples from the western population (*S.t.dresseri*) and seven samples from the eastern population (*S.t.iranica*) (Fig. 2B). Two eastern-western major clades with high support were identified through both NJ and Bayesian analysis (Fig. 2, Suppl. material 1: fig. S2). All *S.t.dresseri* samples formed a strongly supported clade, which is the sister group to another well-supported clade containing samples from the eastern population (Fig. 2A). This differentiation of haplotypes into two clades in the NJ tree was also mirrored by the presence of two haplotype groups in the *S.tephronota* network. In the haplotype network two haplotype groups were separated by 13 base pairs (Fig. 2C). The pairwise F_ST_ and genetic distances between eastern-western populations of *S.tephronota* were 0.91 and 4.1%, respectively (Table 2, Suppl. material 1: table S3). Furthermore, one individual labeled as *S.neumayer* in GenBank (accession number FJ465360.1) and another identified as *S.neumayer* in a GenBank BLAST search both cluster with an eastern subclade of *S.tephronota*.

**Table 2.** Pairwise F_ST_ values between the studied subspecies and species of *S.tephronota* and *S.neumayer* estimated from the mitochondrial data.

Subspecies	*S.t.iranica*	*S.t.dresseri*	*S.neumayer*
***S.t.dresseri***	0.91		
***S.neumayer***	0.93	0.95	
***S.neumayer* (potential admixed)**	0.50	0.96	0.97

*Currucacurruca* is thought to have three breeding subspecies in Iran, including *minula*, *althaea*, and *curruca* and one non-breeding subspecies halimodendri Sarudny, 1911. For this species, we primarily analyzed ten samples from the eastern population and six samples from the western population (Fig. 3B). Two major eastern-western clades with high support were identified through both NJ and Bayesian analysis (Fig. 3, Suppl. material 1: fig. S2). Furthermore, the eastern clade (including samples from Khorasan province) is divided into two well-supported subclades. One eastern subclade comprises individuals from the Dargaz region, a high-elevation breeding area. These samples clustered together with strong support (bootstrap 98%) and, when analyzed alongside *C.c.althaea* birds obtained from GenBank (three individuals), confirmed their taxonomic identity (Suppl. material 1: fig. S3).

Another eastern subclade includes individuals from Sarakhs, a lower-elevation area near the Turkmenistan border, which are strongly separated from another eastern birds (BB 100%) (Fig. 3A). By adding sequences of *C.c.halimodendri* obtained from GenBank (three individuals from Hormozgan province) they are grouped as sister taxa with unresolved relationship (Suppl. material 1: fig. S3). Additionally, another major, well-supported western clade contains samples from the western part of Iran, within the distribution range of *C.c.curruca* (Fig. 3A). In the haplotype network, these two east-west populations were separated from each other by 21 base pairs (Fig. 3C). The pairwise F_ST_ and genetic distances between *C.curruca* subspecies from eastern and western populations in Iran are as follows: *C.c.curruca*/*C.c.althaea* (F_ST_ = 0.99; K2P = 7.04%), *C.c.althaea*/new subclade (F_ST_ = 0.96; K2P = 1.20%), new subclade/*C.c.curruca* (F_ST_ = 1.00; K2P = 6.99%), *C.c.halimodendri*/*C.c.curruca* (F_ST_ = 0.95; K2P = 6.35%), *C.c.halimodendri*/new subclade (F_ST_ = 0.7; K2P = 0.96%) and *C.c.halimodendri*/*C.c.althaea* (F_ST_ = 0.65; K2P = 1.02%) (Table 3 and Suppl. material 1: table S4, respectively).

**Table 3.** Pairwise FST values between the studied subspecies and species of Currucacurruca estimated from the mitochondrial data.

Subspecies	*C.curruca* ssp? (east)	*C.c.curruca* (west)	*C.c.althaea*
***C.c.curruca* (west)**	1.00		
***C.c.althaea* (east)**	0.96	0.99	
***C.c.halimodendri***	0.70	0.95	0.65

*Cardueliscarduelis* and *C.caniceps* are distributed in west and east of Iran respectively (Gill et al. 2024). For these two species, 17 different samples from the western species (*C.carduelis*) and nine samples from the eastern species (*C.caniceps*) were analyzed (Fig. 4B). These samples formed two main eastern-western clades; however, they received insufficient support (Fig. 4A). In the haplotype network, these two east-west populations were separated by two base pairs (Fig. 4C). Furthermore, one individual sampled from the west of Iran (Yasuj) was located in the eastern clade in both the phylogeny and the haplotype network. The pairwise F_ST_ and genetic distances between *C.carduelis* and *C.caniceps* were 0.71 and 0.66%, respectively.

### ﻿Patterns of COI gene variation

**Figure 5.**
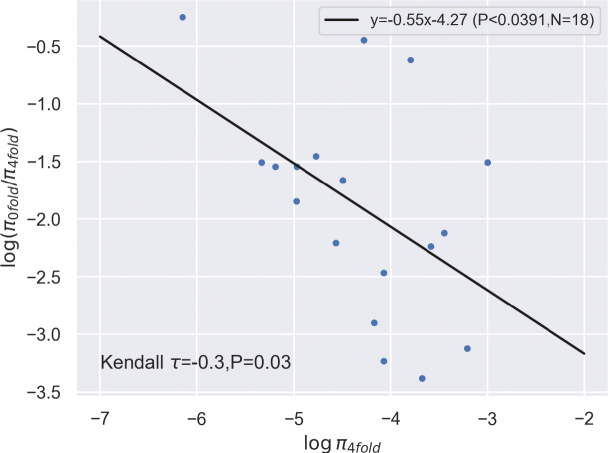
The relationship between log (π _0-fold/π4-fold_) and log (π _4-fold_) *COI* sequences measured for 18 species. The two measures are significantly correlated. The coefficients and p-values of a linear regression as well as Kendall’s rank correlation are shown.

We quantified site-specific coding diversity (i.e., at 0-fold and 4-fold degenerate sites) of each species where we had multiple samples (Suppl. material 1: table S5). For subsequent analysis, we excluded all species with zero diversity at either of these site types and found for all remaining species that the logarithmic ratio of π 0-fold and π 4-fold is negatively correlated with π 4-fold (Fig. 5) which is consistent effective population size scaled effectiveness of selection (James et al. 2016)

## ﻿Discussion

### ﻿DNA library for passerine bird species

Here, we provide a DNA barcode reference library for a substantial dataset of passerine birds in Iran, encompassing the identification of 94 distinct species. This study provides an important foundation for understanding the genetic diversity of Iranian passerine birds through DNA barcoding, with extensive sampling from both eastern and western regions. Our results again demonstrate that DNA barcoding is an effective tool for preliminary biodiversity assessments. No species shared sequences or had overlapping clades with any other species, and every passerine species had distinct *COI* sequences. The development of this DNA barcode library provides a valuable resource for the biodiversity of passerine birds in Iran and will facilitate future studies on the geographic variation and genetic diversity of passerine birds in this area. Our results generally do not resolve phylogenetic relationships above the generic level for Fringillidae, Emberizidae, and Muscicapidae, at the generic level for *Luscinia* and *Emberiza* and, at the species level for *L.collurio*, all of which exhibited paraphyletic patterns in the phylogenetic analysis (Suppl. material 1: fig. S2). Therefore, using only *COI* alone cannot address higher-level taxonomic controversies in some cases. To further clarify the taxonomic uncertainties of higher passerine taxa, multiple nuclear markers using phylogenomic approaches (Zhao et al. 2023), potentially combined with detailed morphological comparisons, are required. Below, we discuss our findings in relation to the genetic diversity of the mentioned challenging taxa in Iran and the influence of DNA barcoding in unrevealing biogeographic patterns.

### ﻿High intraspecific genetic distance and subtle morphological variation

Several DNA barcoding studies of birds have revealed genetically distinct yet morphologically cryptic species (Aliabadian et al. 2013; Saitoh et al. 2015; Bilgin et al. 2016). In our study, we identified two species, *S.tephronota* and *C.curruca*, that may include cryptic species. Both taxa exhibit high intraspecific genetic variation while showing only subtle intraspecific morphological differences. This highlights the challenges in distinguishing cryptic species based solely on traditional morphological methods, as these species may appear similar in morphology but harbor significant genetic divergence (Abdilzadeh et al. 2020). Our results support the subdivision of *S.tephronota* into two major well-supported east (*S.t.dresseri*) – west (*S.t.iranica*) clades (Fig. 2). This genetic split among Iranian populations of *S.tephronota* was first identified by Päckert et al. (2020) in their efforts to clarify the phylogeny of the *Sitta* genus. They included only two samples from Iran, and they discovered a genetic divergence between a sample from eastern Iran (from Dargaz) and an unidentified sample referenced in Pasquet et al. (2014). However, they noted that it remains unclear whether the unidentified sample corresponds to *S.t.dresseri* or *S.t.iranica*. Based on our results, we conclude that this unidentified sample was likely a representative of *S.t.dresseri*, as it clustered with other samples from western Iran.

Moreover, our NJ tree and haplotype network showed that two individuals identified in NCBI as *S.neumayer* were located in the eastern clade of *S.tephronota*. *S.tephronota* has an ecologically and morphologically similar congener, *S.neumayer*, which have overlapping distribution ranges in eastern Turkey and Iran (Mohammadi et al. 2016). These two samples, which were collected from eastern Iran (Khorasan), might be misidentified birds or may represent potential admixed individuals, as it is primarily assumed that hybridization occurs between *S.tephronota* and *S.neumayer* in Iran (Haffer 1977). However, because mtDNA is typically maternally inherited, it is insufficient for identifying interspecific admixed individuals. Biparentally inherited nuclear gene marker is required to complement mtDNA data (Gruenthal and Burton 2005). Therefore, whether these two sister species meet without gene exchange or hybridized to a greater or lesser extent, additional sampling from their potential contact zone is needed. In addition, similar results were found by Elverici et al. (2021), when analyzing mitochondrial *ND2* and *ND3* gene sequences for *S.tephronota* and *S.neumayer*, and they indicated a reciprocal monophyly with no gene flow between birds in the Zagros Mountains and other populations. Furthermore, *S.europaea*, used as an outgroup for *Sitta* phylogenetic tree, exhibits genetic divergence between our samples from the western/northwestern population in Iran (*S.e.persica* and/or *S.e.rubiginosa*) and two other samples representative of the European haplotype. This finding aligns with previous results, which identified two additional Caspian mitochondrial lineages of *S.europaea* from Iran (Nazarizadeh et al. 2016).

The *C.curruca* complex is an intricate model for studying cryptic speciation, presenting challenges in taxonomy due to conflicting morphological and genetic data (Abdilzadeh et al. 2020). Our analysis revealed a basal genetic split in this species between eastern (*C.c.althaea*) and western (*curruca*) subspecies in Iran, supporting previous findings (Olsson et al. 2013; Abdilzadeh et al. 2020; Abdilzadeh et al. 2023). Abdilzadeh et al. (2023) suggested that *C.c.caucasica*, *C.c.zagrossiensis*, and *C.c.curruca* occur in western Iran, and that all are synonyms due to their phylogeographic clustering. Our phylogenetic trees indicate two well-supported subclades within the eastern clade: one consisting of *C.c.althaea*, breeding in eastern Iran (Dargaz), and another sister subclade from lowland northeastern Iran (Sarakhs). However, northeastern Iran is considered part of the distribution range for *althaea* and potentially *C.c.halimodendri* or *C.c.minula* (Shirihai et al. 2001; Votier et al. 2016; Clement 2023). Nonetheless, there remains limited consensus regarding the presence of *C.c.halimodendri* and *C.c.minula* in this region. The only genetic material from this region comes from the study by Abdilzadeh et al. (2020) on samples from the Dargaz region, which were identified as *althaea* birds. It is unlikely that this new subclade represents *minula*, as the new eastern subclade is positioned as a sister group to *althaea*. This contradicts the findings of Olsson et al. (2013), who identified *minula* as a sister taxon of *curruca*. *Currucac.halimodendri* is another suggested taxon, which is assumed to occur in northeastern Iran (Olsson et al. 2013). Three sequences of *C.c.halimodendri* deposited in GenBank were all collected outside of the breeding season from Hormozgan province (Abdilzadeh et al. 2020). Based on the phylogenetic analysis, which included our samples from Sarakhs (new eastern subclade) and three *C.c.halimodendri* samples from southeastern Iran deposited in GenBank, this new subclade is positioned as a sister subclade with *C.c.althaea*. However, the relationships between *C.c.halimodendri* and this new subclade remain unresolved (Suppl. material 1: fig. S3). Furthermore, this new subclade exhibits lower genetic distance and genetic differentiation with *C.c.halimodendri* compared to *C.c.althaea* (Table 3, Suppl. material 1: table S4). Nevertheless, due to the lack of *C.c.minulaCOI* sequences in GenBank and the limited sampling from northeastern Iran, we propose that this new subclade represents a sister taxon to *C.c.halimodendri* and *C.c.althaea*. However, additional studies, including broader geographic sampling and genetic data, are required to determine whether this new subclade represents a distinct population or a previously unrecognized taxon.

### ﻿Low intraspecific genetic distance and high morphological variation: a case study of *Cardueliscarduelis* and *Cardueliscaniceps*

The results revealed a split between two phenotypically different species *C.carduelis* and *C.caniceps*. *Cardueliscarduelis* ranges into the Zagros mountains in the west and north of Iran, whereas on the eastern side of its distributional range in Iran, it is replaced by the morphologically divergent species, *C.caniceps*, which ranges further north into south-central Siberia and northwestern Mongolia (Gill et al. 2024). These two species show substantial differences in the plumage coloration and ornaments and there are some conflicts regarding their classification. For example, Dickinson and Christidis (2014) and Clements et al. (2023) consider the taxon *caniceps* as a subspecies group within *C.carduelis* whereas Gill et al. (2024) consider these two taxa as separate species (i.e., *C.carduelis* and *C.caniceps*). In the NJ tree, the eastern (*C.caniceps*) and western (*C.carduelis*) species formed distinct subclades; however, these subclades were weakly supported, with low bootstrap values. While they appear to form monophyletic groups, the low bootstrap support suggests weak phylogenetic resolution (Fig. 4A). Furthermore, the observed intraspecific genetic distance (0.43%) (Suppl. material 1: table S2) is significantly lower than the average interspecific genetic distances in our dataset. Nevertheless, it is suggested that ornaments may hinder gene flow between distinct or partially distinct populations if shaped by ecological differences or reinforcement, and this ability to modify ornaments, through mechanisms like sexual selection or reinforcement, could influence the formation of new species over time (Cardoso and Mota 2008).

### ﻿Effect of selection on COI gene

According to population genetic theory, the effectiveness of selection is more pronounced in species with larger effective population sizes (Woolfit 2009; Gossmann et al. 2010, 2012). Here we tested the effect of selection on protein coding mutations in the *COI* gene by contrasting the genetic diversity of mutations that change the amino acid at 0-fold degenerate sites versus those mutations that are silent at 4-fold degenerate sites (James et al. 2016). Species with larger effective population sizes should show relatively fewer amino-acid changing mutations because the selection is more efficient in larger populations. We were able to obtain non-zero coding diversity measures for 18 species and find a correlation between log (π 4-fold/0-fold) versus log (π 0-fold). The slope is negative and highly significant which suggests that much of the genetic variation is consistent with population size scaled effects of purifying selection and drift. Our results also suggest that much of the observed variation stems from mutations at synonymous sites.

### ﻿Biogeographical aspects

The current distribution and genetic makeup of species in Iran reflect its unique biogeographic characteristics (Ficetola et al. 2017; Yusefi et al. 2019). The region’s transitional geographic position is exemplified by its diverse assemblage of animal species from distinct biogeographic zones. From the Palearctic realm, notable species include the Red deer (*Cervuselaphus*), Roe deer (*Capreoluscapreolus*), Brown bear (*Ursusarctos*), Eurasian lynx (*Lynxlynx*), European green woodpecker (*Picusviridis*), Tawny owl (*Strixaluco*), and Meadow viper (*Viperaeriwanensis*). The Saharo-Arabian zone contributes species such as gazelles (*Gazellasubgutturosa*, *G.bennettii*, *G.gazella*), the Cheetah (*Acinonyxjubatus*), Sand fox (*Vulpesrueppellii*), Desert cobra (*Walterinnesiaaegyptia*), and Black-striped hairtail butterfly (*Antheneamarah*). From the Oriental realm, the region hosts the Asiatic black bear (*Ursusthibetanus*), Palm squirrel (*Funambuluspennanti*), Indian crested porcupine (*Hystrixindica*), Persian krait (*Bungarussindanuspersicus*), Bay-backed shrike (*Laniusvittatus*), Sykes’s nightjar (*Caprimulgusmaharattensis*), Striped Pierrot butterfly (*Tarucusnara*), and Baphomet moth (*Creatonotosgangis*) (Noori et al. 2024). In addition to its role as a biogeographical transition zone, Iran’s mountainous topography has played a pivotal role in shaping species distributions and genetic patterns by acting as a barrier and/or corridor, or as glacial refugia during the Pleistocene (Moradi et al. 2024). Mountain ranges such as the Alborz in the north and the Zagros in the west have acted both as barriers to gene flow and as refugia during glaciation periods, contributing to the current genetic differentiation of species. Similarly, the Kopet-Dag in the northeast and the Makran range in the southeast have further limited the distribution ranges of many taxa across the Iranian Plateau, enhancing the biogeographic and genetic diversity of numerous genetic lineages and had a profound impact on the patterns of inter- and intraspecific variation among species (Rajaei et al. 2013; Hosseinzadeh et al. 2020). This biogeographic structure in Iran is even more pronounced in animals with low dispersal abilities and narrow ecological niches, such as reptiles. For example, biogeographic analyses of the snake fauna reveal three distinct groups: one linking the western Zagros and Khuzestan fauna with the Saharo-Arabian region, a second connecting the Kopet Dagh and Turkmen Steppe fauna with the Turanian region, and a third associating the Central Plateau and Baluchistan fauna with the Iranian region (Moradi et al. 2024).

In this study, the patterns of intraspecific divergence observed in *S.tephronota*, *C.curruca*, *C.carduelis*, and *C.caniceps* align with Iran’s key zoogeographic boundaries and geographical barriers. These patterns reflect an east-west geographical split within these species that corresponds to the distinct biogeographical realms they inhabit. This highlights the influence of Iran’s transitional geographic position, as well as the role of mountains and refugia, in shaping species differentiation and current genetic patterns. In addition, this east-west genetic distinctiveness in our target taxa is paralleled in several other widespread sister Eurasian passerine taxa that have allopatric populations at their southern range margin in Iran, such as the Eurasian nuthatch, *S.europaea* (Nazarizadeh et al. 2016; Päckert et al. 2020), coal tit, *Periparusater* (Tietze et al. 2011), horned lark, *Eremophilaalpestris* (Ghorbani et al. 2020) and great tit, *Parusmajor* (Javaheri Tehrani et al. 2021).

## ﻿Conclusions

The present study fills a significant biodiversity knowledge gap in the barcoding data of passerine birds in Iran and demonstrates the utility of standardized DNA-based species delimitation methods in enhancing biodiversity inventories. The observed patterns of intraspecific divergence in *S.tephronota*, *C.curruca*, *C.carduelis*, and *C.caniceps* align with key zoogeographic boundaries in Iran, reflecting an east-west geographical split within these species. This finding underscores the important role of DNA barcodes in revealing phylogeographical patterns, consistent with previous studies that highlight the effectiveness of DNA barcoding in resolving such patterns (Saitoh et al. 2015; Wu et al. 2023). These examples further highlight Iran’s pivotal role as a biogeography crossroad for avian diversity, with paired species consisting of a western (European or Mediterranean) member and an eastern (Asian) member (Haffer 1977; Roselaar and Aliabadian 2007). These findings collectively underscore the complex impact of Iran’s topography and climatic history on shaping present-day avian genetic diversity (Noori et al. 2024).

## ﻿Additional information

### Author ORCIDs

Sahar Javaheri Tehrani https://orcid.org/0000-0002-4824-1837

Elham Rezazadeh https://orcid.org/0000-0003-4935-0522

Leila Nourani https://orcid.org/0000-0001-7932-2480

Asaad Sarshar https://orcid.org/0000-0002-4325-6706

Toni I. Gossmann https://orcid.org/0000-0001-6609-4116

Mansour Aliabadian https://orcid.org/0000-0002-3200-4853

### Data availability

Sequence data and their accession numbers have been deposited in the NCBI database (https://www.ncbi.nlm.nih.gov).

## Supplementary materials

Supplementary material 1 Additional information Data type docx Explanation note **fig. S1.** Sampling locations. Colors and the size of circles represents the number of individuals. **fig. S2.** Bayesian tree of *COI* sequences from 96 species of Iranian passerine birds. Values at nodes show posterior probabilities; full support is indicated with an asterisk. Species that form reciprocally monophyletic clades have been collapsed. Two species showed deep intraspecific divergence: (a) *Currucacurruca* and (b) *Sittatephronota*; these are marked in blue. Families labeled on the right of the figure. **fig. S3.** Phylogenetic and haplotype network analysis of *COI* data for *C.curruca*. a) Neighbor-joining tree, values on the branches shows bootstrap values and, our *COI* sequences are indicated in red. b) Haplotype network, where colors indicate the origin of the haplotypes (Orange: western population; Green: eastern populations) and the number of bars at each branch indicates the number of mutations. **table S1.** List of all Iranian passerine birds that have been sequenced in this study, with voucher numbers and collection localities. Coordinates are given in decimal degrees. **table S2.** Comparisons of K_2_P-pairwise distances within species. Distances are calculated for Iranian passerine birds for which two or more sequences were available; species including one individual are not calculated (n/c); three challenging taxa indicated bold and highlighted grey; distances are expressed in percentages. **table S3.**K2P distances (%) for the populations of *S.tephronota* and *S.neumayer* (*COI* seq), below the diagonal between group average. **table S4.**K2P distances (%) for the populations of *Currucacurruca* (*COI* seq), below the diagonal between group average. **table S5.**K2P distances (%) for the populations of *Cardueliscarduelis* (*COI* seq), below the diagonal between group average. **table S6.** Genetic diversity at coding sites. Non-synonymous nucleotide diversity πn, number of non-synonymous nucleotide diversity N, synonymous nucleotide diversity πs, number of synonymous nucleotide diversity S. https://binary.pensoft.net/file/1314742This dataset is made available under the Open Database License (http://opendatacommons.org/licenses/odbl/1.0/). The Open Database License (ODbL) is a license agreement intended to allow users to freely share, modify, and use this Dataset while maintaining this same freedom for others, provided that the original source and author(s) are credited.Sahar Javaheri Tehrani, Elham Rezazadeh, Niloofar Alaei Kakhki, Leila Nourani, Vali Ebadi, Sahar Karimi, Mojtaba Karami, Fatemeh Ashouri, Asaad Sarshar, Toni I. Gossmann, Mansour Aliabadian
